# The Nox2 NADPH oxidase regulates neutrophilic inflammation in the oral cavity

**DOI:** 10.1016/j.mucimm.2026.04.002

**Published:** 2026-04-08

**Authors:** Shunying Jin, Richa Singhal, Jianzhu Luo, Yasmine H. Higgins, Kelley N. Cooper, Marina Terekhova, Katherine A. Carey, Audrey F. Duff, Maxim N. Artyomov, Venkatakrishna R. Jala, Rachel A. Idol, Michael T. Bailey, Mary C. Dinauer, Richard J. Lamont, Juhi Bagaitkar

**Affiliations:** a Department of Oral Immunology and Infectious Diseases, University of Louisville, Louisville, KY, United States; b Center for Microbe and Immunity Research, Abigail Wexner Research Institute, Nationwide Children’s Hospital, Columbus, OH, United States; c Department of Pathology and Immunology, Washington University School of Medicine, St. Louis, MO, United States; d Department of Microbiology and Immunology, Brown Cancer Center, School of Medicine, University of Louisville, Louisville, KY, United States; e Department of Pediatrics, Washington University School of Medicine, St. Louis, MO, United States; f Department of Pediatrics, College of Medicine, The Ohio State University, Columbus, OH, United States; g These authors are contributed equally.

## Abstract

The leukocyte NADPH oxidase 2 (Nox2) is an important regulator of inflammatory responses, independent of its antimicrobial activity. Inactivating mutations in NOX2 cause chronic granulomatous disease (CGD), a severe immunodeficiency associated with recurrent infections and dysregulated neutrophilic inflammation. Recurrent oral ulcers, stomatitis, gingivitis, and other inflammatory issues affecting the oral mucosa have been observed in patients with CGD; however, the underlying mechanisms are not known. Here, we present evidence that the extensive inflammatory destruction of oral mucosal tissues observed in Nox2-deficient or *Cybb*^*KO*^ mice was not caused by impaired antimicrobial surveillance against oral pathobionts but instead resulted from a cell-intrinsic dysregulation of neutrophil inflammatory responses. Transcriptional and cellular profiling of oral tissues isolated from wild-type and *Cybb*^*KO*^ mice showed a dominant neutrophil signature, which was accompanied by a significant upregulation of several bone-resorbing, tissue-degrading inflammatory cytokines and a reduced expression of nuclear factor erythroid 2-related factor 2 (Nrf2) regulated genes. Mechanistically, hyperinflammatory responses were mitigated by restoring Nrf2 transcriptional activity using a synthetic agonist. Thus, our studies show that the Nox2 oxidase and derivative reactive oxygen species are crucial for balanced neutrophil recruitment and cell-intrinsic regulation of their inflammatory responses within oral tissues in an Nrf2-dependent manner.

## Introduction

The leukocyte NADPH oxidase 2 (Nox2) is a multi-subunit enzyme complex predominantly expressed in phagocytes. The activation and assembly of the Nox2 complex on plasma or phagosomal membranes generates superoxide $\left({\text{O}}_{2}^{-}\right)$, a precursor to antimicrobial reactive oxygen species (ROS) that have critical immunoregulatory roles. Inactivating mutations in the Nox2 oxidase subunit genes are associated with chronic granulomatous disease (CGD), a severe immunodeficiency characterized by life-threatening infections and an excessive inflammatory response^1,2^. Dysregulated inflammatory responses are a significant aspect of CGD pathology, and patients frequently present with sterile granulomatous inflammation in the lungs, gastrointestinal tract, and mucocutaneous skin lesions. Interestingly, patients with CGD are also prone to oral inflammation, characterized by stomatitis, recurrent oral ulceration affecting the cheeks, lips, and floor of the mouth^3–5^, and gingival inflammation^6^. Despite the prevalence of oral mucosal inflammation in patients with CGD, insights into the underlying mechanisms are lacking.

Nox2 is highly expressed in neutrophils, a cell type highly abundant in oral mucosal tissues. Neutrophils are actively and continuously recruited into the oral cavity, a hostile environment rich in diverse activating stimuli including bacterial and fungal ligands, food antigens, allergens, and ongoing tissue damage due to mastication. Within the gingival tissues, neutrophils limit microbial numbers and barrier breaches by phagocytosing bacteria, degranulating, and forming neutrophil extracellular traps (NETs), thereby playing an important role in antimicrobial surveillance. Their presence is also essential for wound healing and immune homeostasis upon efferocytic clearance^7,8^. However, the cell-intrinsic mechanisms that restrain neutrophil effector responses in the oral cavity to prevent tissue damage via excessive activation under homeostatic conditions remain unknown. A substantial body of literature provides compelling evidence that Nox2-derived ROS, independent of their antimicrobial role, play a vital role in modulating neutrophil inflammatory potential within tissues^9^. Interestingly, neutrophils lacking Nox2 exhibit a primed or activated phenotype with enhanced azurophilic granule exocytosis, coupled with excessive release of tissue-degrading proteases^10^. The absence of Nox2 activity was also associated with elevated calcium entry in neutrophils and increased levels of the inflammatory lipid mediator leukotriene B4^11,12^. Other studies demonstrate that excessive activation of the transcription factor NF-κB (Nuclear factor-kappa B) led to overproduction of many pro-inflammatory cytokines and higher inflammasome activity^13,14^ that correlated with increased IL-1 production in neutrophils and monocytic cells^15–17^. Furthermore, we previously showed that Nox2 deficiency was also linked to the excessive mobilization of neutrophils from bone marrow pools, resulting in their increased accumulation within the inflamed tissues of Cybb/Nox2 knockout (*Cybb*^*KO*^) mice, resulting in a significant delay in resolving the inflammatory response^16–20^. Thus, Nox2 and derivative ROS have been unequivocally linked with the regulation of neutrophil inflammatory responses.

Here, we examined whether Nox2 and its derivative ROS control inflammatory responses at the oral mucosa, a barrier surface heavily populated by microbes. Our mechanistic studies in mouse models show significant damaging inflammation in *Cybb*^*KO*^ mice characterized by erosion of oral soft tissues and the recession of alveolar bone, primarily driven by the overabundant recruitment of neutrophils into oral tissues and their hyperactivation. Interestingly, despite the presence of a dysbiotic microbial community, we found no signs of sepsis or systemic dissemination of periodontal pathobionts. The absence of Nox2 led to a profound failure in the activation of nuclear factor erythroid 2-related factor 2 (Nrf2), an essential redox-sensitive transcription factor that also represses inflammatory response. Thus, our findings highlight a previously uncharacterized role for Nox2 in cell-intrinsic regulation of neutrophil responses within oral tissue, an environment replete with varied activating stimuli.

## Methods

### Mice:

We purchased C57BL/6J wild-type (WT) mice from the Jackson Laboratory. Dr. Mary Dinauer provided Cybb/Nox2 knockout (*Cybb*^*KO*^) mice^21^ and conditional knockout mice lacking Nox2 activity in neutrophils (*Cybb*^f/f^ *Ncf2*^f/f^)^22^. Dr. Krishna Jala provided Nrf2 knockout (*Nfe2l2*^*−/−*^) mice. Both male and female mice, aged 6–10 weeks, were used for all studies to account for sex as a biological variable. The Institutional Animal Care and Use Committees (IACUC) at the University of Louisville and the Abigail Wexner Research Institute at Nationwide Children’s Hospital approved all experiments.

### Ligature model of oral inflammation:

Gingival inflammation was induced by placing a 5–0 silk ligature around the second maxillary molar of mice^23^. After 8 days, the mice were euthanized, and the gingival tissues surrounding the ligatures were isolated and either flash-frozen for RNA extraction or enzymatically digested for flow cytometry^24^. Mouse heads were fixed in 10% formalin and scanned using micro-computed tomography (μCT). Average bone loss was measured by taking linear measurements (in millimeters) from the cementoenamel junction (CEJ) to the alveolar bone crest (ABC) in the interdental regions between the first and second molars (M1-M2) or the second and third molars (M2-M2′) (Fig. S1A–B) and represented as CEJ-ABC length, as previously described by Park et al.^25^ In specific experiments, mice received 25 mg/kg sulforaphane (SFN) intraperitoneally (i.p.) once daily for 7 days after ligature placement^26^. For experiments related to microbiome analysis, female WT and *Cybb*^*KO*^ mice were co-housed for a month prior to ligature placement.

### Bacteria:

*Porphyromonas gingivalis* 33,277 was cultured anaerobically in enriched trypticase soy broth (eTSB) supplemented with hemin (5 μg/mL), menadione (1 μg/mL), and 1 mg/mL yeast extract. *Fusobacterium nucleatum* 25,866 was cultured anaerobically in brain–heart infusion broth (BHI) with yeast extract (1 mg/mL), hemin (5 μg/mL), and menadione (1 μg/mL). *Filifactor alocis* was cultured anaerobically in BHI with yeast extract (1 mg/mL), hemin (5 μg/mL), menadione (1 μg/mL), cysteine (0.05%), and arginine (0.05%). All strains were grown at 37 °C with 85% N_2_, 10% H_2,_ and 5% CO_2_. Ligatures were isolated from WT and *Cybb*^KO^ mice, placed in 1 mL of sterile phosphate-buffered saline (PBS), and vortexed to dissociate ligature-associated bacteria. Suspension (0.5 mL) was added to two tubes containing 10 mL of eTSB to cultivate ligature-associated bacteria and then incubated either aerobically or anaerobically overnight. The next day, 10^7^ CFU of aerobically and 10^7^ CFU anaerobically cultured bacteria were combined in PBS and injected into mice i.p.

### Bacterial peritonitis:

Mice were challenged with either 10^7^ colony-forming units (CFUs) of *P. gingivalis* 33,277 or 10^7^ CFU of ligature-associated oral bacteria (i.p.) and euthanized after 4 h. Peritoneal cavities were lavaged with 4 ml sterile PBS + 2 mM ethylenediaminetetraacetic acid (EDTA). Lavage fluid (50 μL) was plated on trypticase soy broth (TSB) blood agar plates to estimate bacterial CFUs (in vivo survival). Separately, lavage fluid was analyzed for inflammatory cytokines by 32-plex cytokine arrays, and leukocyte differentials were determined by flow cytometry.

### Degranulation assays:

Degranulation of mouse bone marrow neutrophils (BMNs) was measured by assessing the mobilization of granule markers to the plasma membrane by flow cytometry. BMNs were challenged with various bacteria at a multiplicity of infection of 1:10 and 1:100 (cell: bacteria) at the indicated time points. At each time point, cells were washed twice with 3 mL of ice-cold flow buffer (1% bovine serum albumin, 2 mM EDTA in PBS). For flow staining, cells were resuspended in 50 μL of 2.4 G2 supernatants to block Fc receptors for 10 min at room temperature and then stained with the fluorochrome-conjugate anti-mouse Ly6G-V450, CD63-PE, and CD35-BV605 antibodies for 1 h on ice. Data were collected on FACS Celesta (BD Biosciences) and analyzed with FlowJo V10.

### Data availability:

RNA sequencing data have been submitted to the Gene Expression Omnibus database under Accession No. GSE184556. The metagenomic data are in SRA PRJNA1235937.

### Statistical analysis:

GraphPad Prism, Version 9.3.1, was used for all statistical analyses. A detailed description of the specific statistical test used for data analysis, as well as post-hoc correction, is provided in the legend of each figure.

**Please see Supplemental Materials for additional methods.

## Results

### Nox2 deficiency enhances neutrophil recruitment and inflammatory damage in oral tissues

**Fig. 1.**
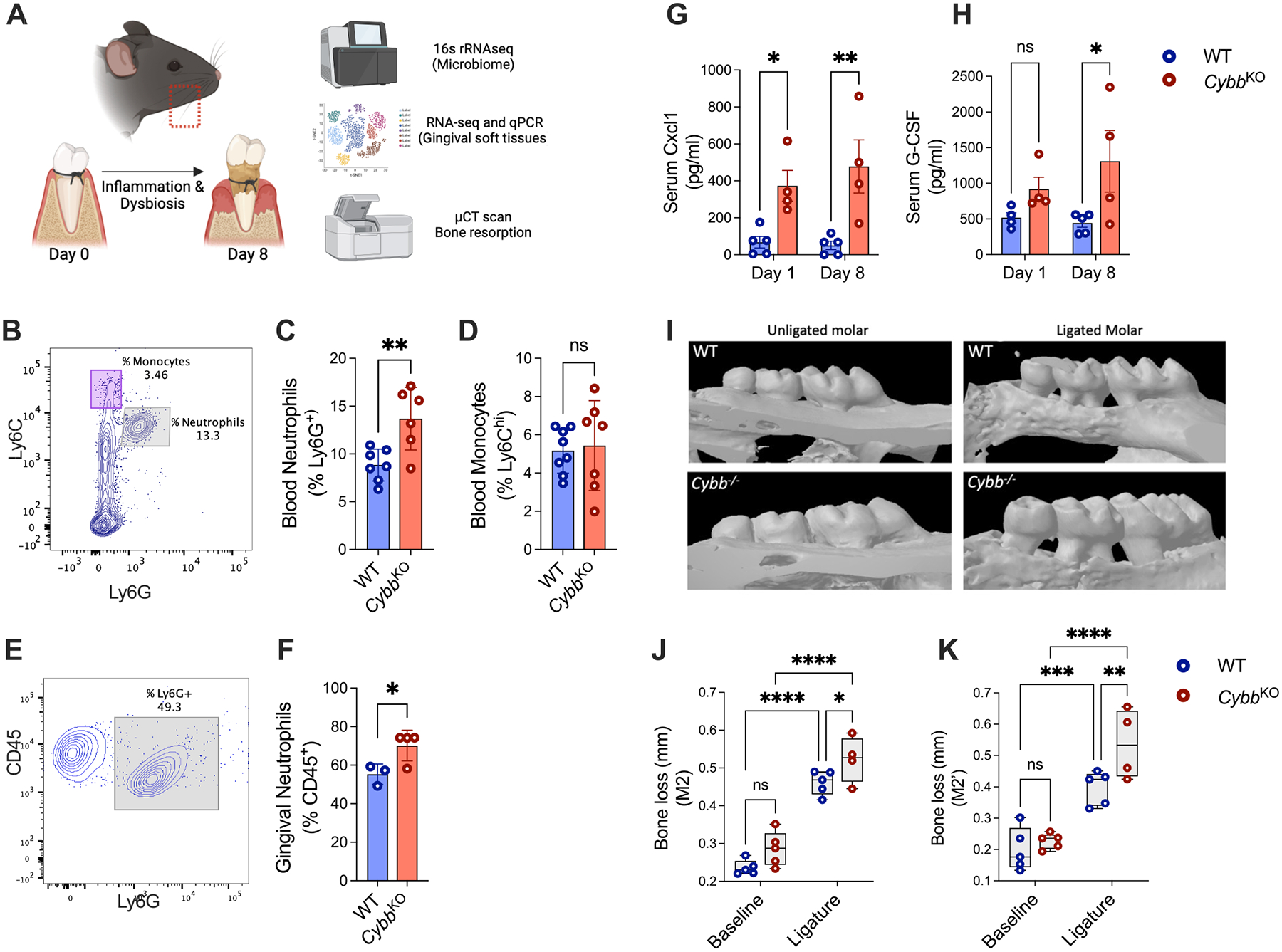
Nox2 deficiency drives excessive neutrophil recruitment and damage in oral mucosal tissues. (**A**) Illustration depicting the ligature-induced periodontitis in mice on the left second maxillary molars of wild-type (**WT**) and *Cybb*^*KO*^ mice. (**B-D**) 24 h after ligature placement, the relative abundance of neutrophils (Ly6G^hi^ Ly6C^int^) and monocytes (Ly6C^hi^ Ly6G^−^) were determined in peripheral blood via flow cytometry. (**E-F**) Gingival neutrophil numbers on Day 8. Data from 4 to 7 mice per group are displayed as mean ± SD, and a *t*-test determined statistical differences (*P < 0.05; **P < 0.01). (**G-H**) Multiplexed 32-cytokine arrays determined serum levels of KC/CXCL1 and granulocyte colony-stimulating factor (G-CSF) on Day 1 and Day 8 post-ligature placement. (**I–K**) Average bone loss (in millimeters) in the interdental regions (M2, M2′) of the ligated molar and unligated contralateral control molars from the same mice. Data from 5 to 8 mice per group are displayed as mean ± SD, and statistical differences were determined using two-way ANOVA with Sidak’s multiple comparison tests (*P < 0.05; **P < 0.01; ***P < 0.001; ****P < 0.0001).

To reproducibly model oral inflammation in mice, we used the well-characterized ligature-induced periodontitis (LIP)^23^. In this model, ligature placement around the second maxillary molar leads to microbial accumulation, immune cell infiltration, and localized bone resorption (Fig. 1A). We observed a significantly higher mobilization of neutrophils into the blood within 24 h of ligature placement in Nox2/Cybb knockout (*Cybb*^KO^) mice^21^, which tracked with significantly higher neutrophil accrual in the gingival tissues compared to WT mice (Fig. 1 B–F). To determine the underlying mediator driving neutrophil mobilization, we performed a multiplexed 32-cytokine array on mouse sera collected post-ligature placement. Our data show an early spike in factors linked with neutrophil mobilization, such as granulocyte colony-stimulating factor and CXCL1 (KC)^27^ that remained high until Day 8 in the blood of *Cybb*^KO^ mice, but not WT mice (Fig. 1G–H). This was consistent with our previous observations in models/tissues, such as sterile peritonitis^16^ or zymosan-induced lung injury^17^, where the absence of Nox2 activity was associated with an unbalanced neutrophil response to experimentally induced inflammation.

Excessive neutrophil recruitment and their prolonged activation have been shown to contribute significantly to localized inflammatory bone loss in the oral cavity^28^. Dysregulated neutrophil responses not only lead to soft tissue ulceration but have been shown to be associated with inflammatory bone recession in mice^23,29^ and in patients with neutrophil-related immunodeficiencies^30^. In the LIP model, μCT analysis of mouse skulls on Day 8 demonstrated pathological resorption of alveolar bone around the ligated molars, which was significantly more severe in *Cybb*^KO^ mice (Fig. 1I–K). Average bone loss was measured by taking linear measurements (in millimeters) from the CEJ to the ABC in the interdental regions around the ligature, between the first and second molars (M1-M2) and the second and third molars (M2-M2′) as illustrated and quantified in Fig. S1 A–B.

**Fig. 2.**
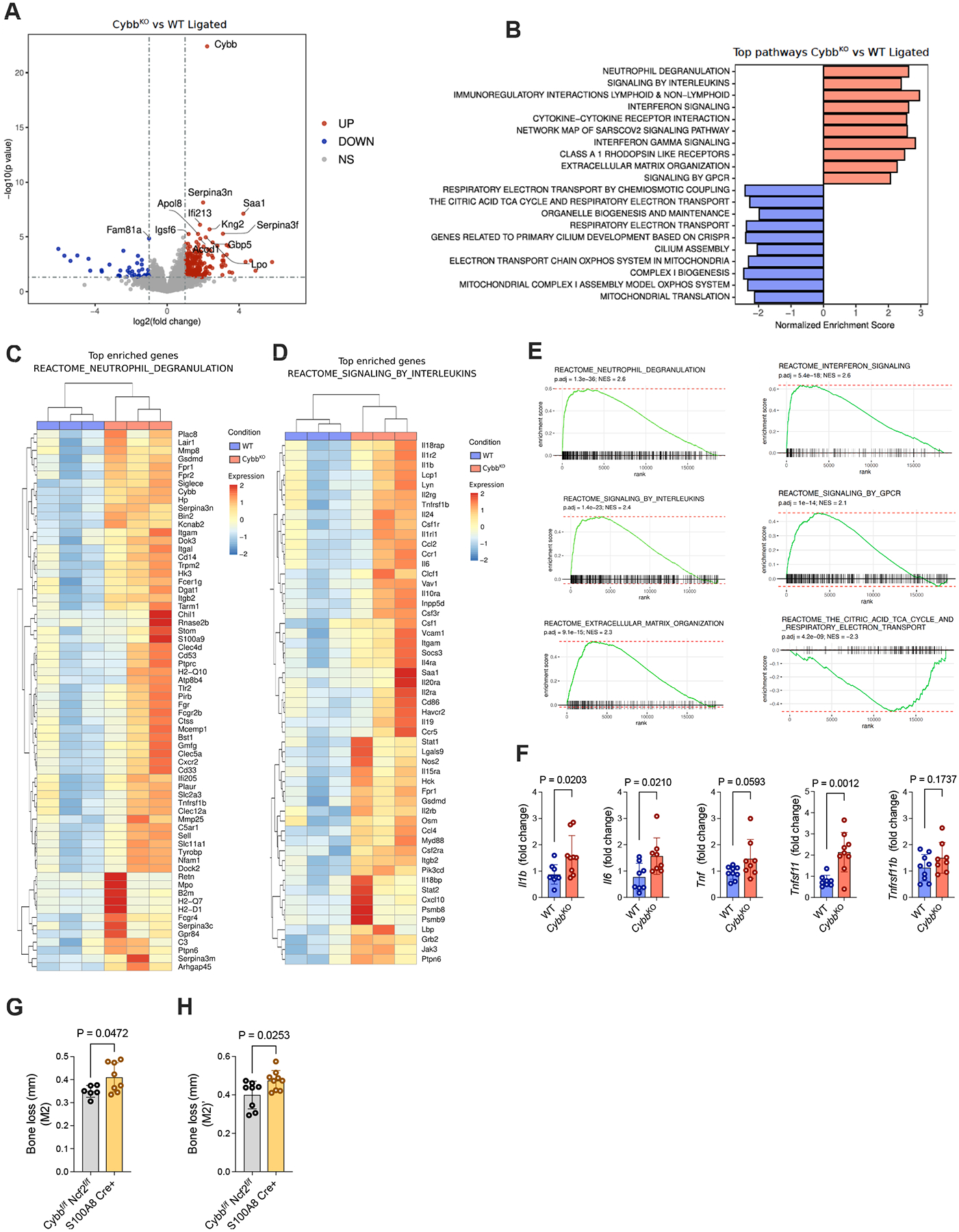
Hyperinflammatory gene signatures predominate in inflamed gingival tissues of Nox2-deficient (*Cybb*^*KO*^) mice. Gingival tissues of wild-type (**WT**) and *Cybb*^*KO*^ mice were extracted on Day 8 and analyzed by RNA-seq. (**A**) Plotting log2 (fold change) versus −log10 (adjusted p-value) generated volcano plots of RNA-seq data, representing differences between ligated WT and *Cybb*^*KO*^. (**B**) Differentially expressed genes (**DEGs**) were used to determine enriched and suppressed pathways. Heatmaps of top DEGs in the (**C**) Reactome_Neutrophil_Degranulation and (**D**) Reactome_Signaling_By_Interleukins. (**E**) Gene set enrichment analysis (**GSEA**) plots of the major enriched pathways. (**F**) Validation of select genes by **qPCR** from gingival tissues. Data from 4 to 7 mice per group are displayed as mean ± SD, and statistical differences were determined using *t*-test. (**G-H**) Average bone loss (in millimeters) was measured in *Cybb*^f/f^ *Ncf2*^f/f^ or *Cybb Ncf2* S100A8^Cre+^ littermate controls on Day 8 post ligature in the interdental regions (M2, M2′) of the ligated molar and unligated contralateral control molars from the same mice. Data from 6 to 8 mice per group are displayed as mean ± SD, and statistical differences were determined using a *t*-test.

To further delve into the underlying dysregulated molecular pathways, we performed RNA-seq of the gingival tissues. Volcano plots compared the variation in differentially expressed genes (DEGs) in ligated inflamed gingival tissues (Fig. 2A) and in unligated naïve gingival tissues from WT and *Cybb*^*KO*^ mice (Fig. S1C). Interestingly, no significant changes were noted in gene expression in the oral tissues of naïve WT vs. *Cybb*^*KO*^ mice (Fig. S1C). Furthermore, we did not observe any spontaneous bone loss in the unligated/naive *Cybb*^*KO*^ mice, indicating that ROS deficiency does not predispose to spontaneous infections from naturally occurring oral bacteria in mice (Fig. 1I–K). In contrast, the gingival tissues of ligated/inflamed mice exhibited inflammatory gene signatures with many more DEGs and a stronger inflammatory response in Nox2-deficient animals (Fig. 2A). We performed gene-set enrichment analysis in the order of decreasing log2 fold change in a ranked list of all genes. Gene ontology (GO) analysis indicated that the most significantly enriched terms *(according to q values or NES)* were ‘neutrophil degranulation’ and ‘signaling by interleukins’ (Fig. 2 B–D). The top ten enriched pathways are presented in Fig. 2B.

We also conducted a gene set enrichment analysis (GSEA) and observed that pathways for interferon signaling, G-protein-coupled receptor (GPCR) signaling, and extracellular matrix organization were positively correlated, further corroborating our findings (Fig. 2E). Neutrophils have been previously implicated in actively contributing to inflammatory bone loss through cell-intrinsic expression of several bone-resorptive ligands and produce factors such as IL-17 that bolster the recruitment and activity of other osteoclastogenesis-driving immune cells^28^. Several inflammatory cytokines, such as IL-1, IL-6, and TNF family members, are directly linked with osteoclastogenesis by stimulating the production of bone resorptive factors, including receptor activator of NF-κB (RANK) and its ligand (RANK-L/Tnfsf11)^31^. Quantitative polymerase chain reaction (qPCR) analysis confirmed significantly higher expression of osteoclast-activating cytokines *Il-1b, Il-6, and Tnfsf-11* (RANK-L) in LIP gingival tissues in *Cybb*^*KO*^, concordant with the RNA-seq data (Fig. 2F). Osteoprotegerin (Tnfrsf11b), which acts as a decoy ligand for RANK receptors and moderates their osteoclastic potential, was not differentially expressed between WT and *Cybb*^KO^ mice. Finally, conditionally deleting Nox2 function, specifically in neutrophils, was sufficient to drive excessive alveolar bone recession compared to littermate controls. Thus, our data indicate that the absence of Nox2 aggravates inflammatory responses at the oral mucosal barrier by dysregulating neutrophil recruitment and/or activation (Fig. 2G–H).

### Microbial dysbiosis in Cybb^KO^ mice was not associated with systemic infections

**Fig. 3.**
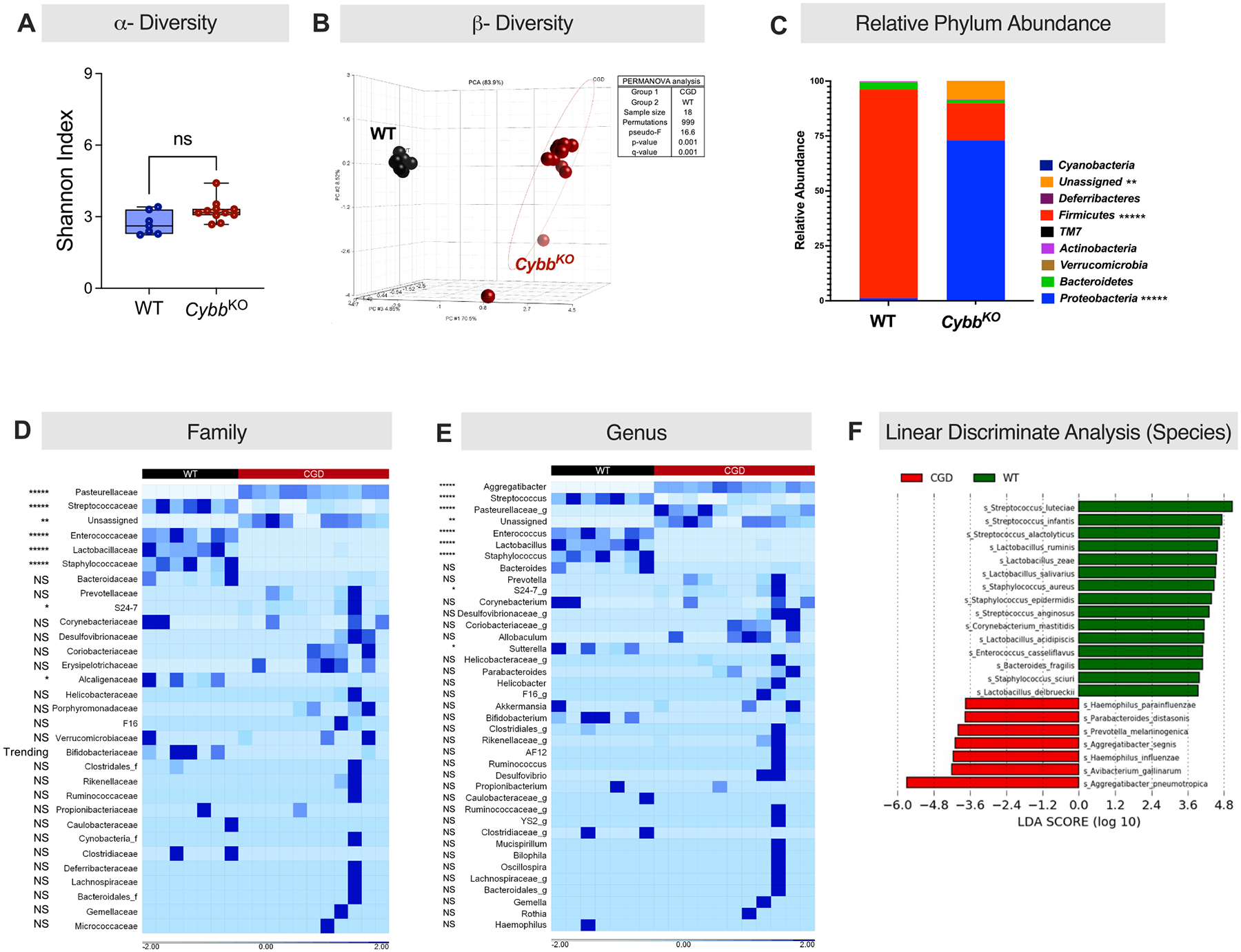
Comparative shifts in oral microbial diversity in the context of Nox2 deficiency during LIP. (**A**) Alpha diversity (species abundance and evenness) was measured with the Shannon diversity index (y-axis). Data represent mean ± SEM for n = 7 for WT and n = 11 for *Cybb*^*KO*^ mice with Mann-Whitney *U* test corrected p-value, ****p < 0.00001. (**B**) Beta diversity (depicting compositional differences) is represented as a three-dimensional Principal Component Analysis (PCA) plot generated using Jaccard similarity matrix. PC1 vs. PC2 indicates a clear separation between *Cybb*^*KO*^ and WT along PC1 (70.5%). PC2 vs. PC3 reveals additional variance (PC2: 8.52%, PC3: 4.85%) and clustering. PERMANOVA analysis was performed to indicate significant compositional differences (q = 0.001). (**C**) Microbial composition at different taxonomic levels: (**C**) Phylum barplot (Left), (**D**) Family heatmap, and (**E**) Genus heatmap. White (light blue) signifies that microbial taxa are present in low abundance or absent, and blue signifies that microbial taxa are highly abundant. Corrected p-value derived (Dunn’s multiple testing) from Mann-Whitney *U* test are depicted as an asterisk next to the microbial taxa: p < 0.05= *, p < 0.01 =**, p < 0.001 =***, p < 0.00001=****, p < 0.000001=*****, NS = not significant, and Trending = p-value between 0.051 and 0.06. **(F**) Linear Discriminate Analysis Effect size representing significant microbial species enrichment in wildtype and *Cybb*^*KO*^ mice. LDA cut off score > 2 and p < 0.05.

Nox2 activation regulates several antimicrobial functions of neutrophils, such as the phagosomal oxidative burst, degranulation, and the formation of neutrophil extracellular traps (NETs)^9^. We investigated whether inadequate immunosurveillance and weakened antimicrobial defense against oral bacteria drove the inflammatory burden in *Cybb*^KO^ oral tissues. The LIP model allows for profiling bacterial biofilms that accumulate around the ligatures to assess species diversity and their collective pathogenic or ‘inflammophilic’ potential in oral tissues^32^. 16S rRNA sequencing of ligature-associated co-housed WT and *Cybb*^*KO*^ mice revealed that the alpha diversity Shannon Index, which measures species richness and evenness within individual samples, did not differ significantly between the groups. However, the beta diversity Jaccard similarity index noted a significant compositional difference between the groups (q-value = 0.001) (Fig. 3A–B).

Taxonomic analysis identified distinctive microbial communities between the groups. We found that the *Cybb*^*KO*^ oral microbiome had increased dominance of the phylum Proteobacteria (typically harboring pathogens) and a concomitant reduction in the phylum Firmicutes (typically harboring beneficial bacteria) compared to WT mice (Fig. 3C). Further taxonomic assessment revealed that within the phylum Proteobacteria, the bacterial family Pasteurellaceae and the genus Aggregatibacter were significantly expanded. At the same time, within Firmicutes, the genera Streptococcus, Enterococcus, Lactobacillus, and Staphylococcus in *Cybb*^*KO*^ mice were significantly reduced (Fig. 3C). To gain further insight into the distinct enrichment profiles between the two groups at the species level, linear discriminant analysis effect size (LEfSe) analyses were performed. An increase in the abundance of Bifidobacterium pseudolongum and other species belonging to Firmicutes distinguished the WT mice from *Cybb*^*KO*^ mice. In contrast, the expansion of Aggregatibacter pneumotropica, Allobaculum, and Pasteurellaceae sp. was a hallmark feature of the microbial communities in the *Cybb*^*KO*^ group (Fig. 3D–F).

Although infections with *Haemophilus* and *Aggregatibacter* have been reported to cause liver abscesses and lymphadenitis in some patients with CGD, these occurrences are relatively rare^33–35^. Typically, patients with CGD are susceptible to life-threatening infections with a narrow spectrum of pathogens. Catalase-positive bacterial pathogens (Staphylococcus aureus, Burkholderia cepacia, Serratia marcescens, Nocardia species) and specific invasive fungal pathogens (*Aspergillus* spp.) cause commonly reported CGD infections^35^. However, recent reports indicate that although rare, specific catalase-negative pathogens such as Chromobacterium violaceum, Francisella philomiragia, and Actinomyces spp can cause significant life-threatening complications in patients with CGD^35^. Thus, we determined whether delayed bacterial clearance contributed to oral inflammation.

**Fig. 4.**
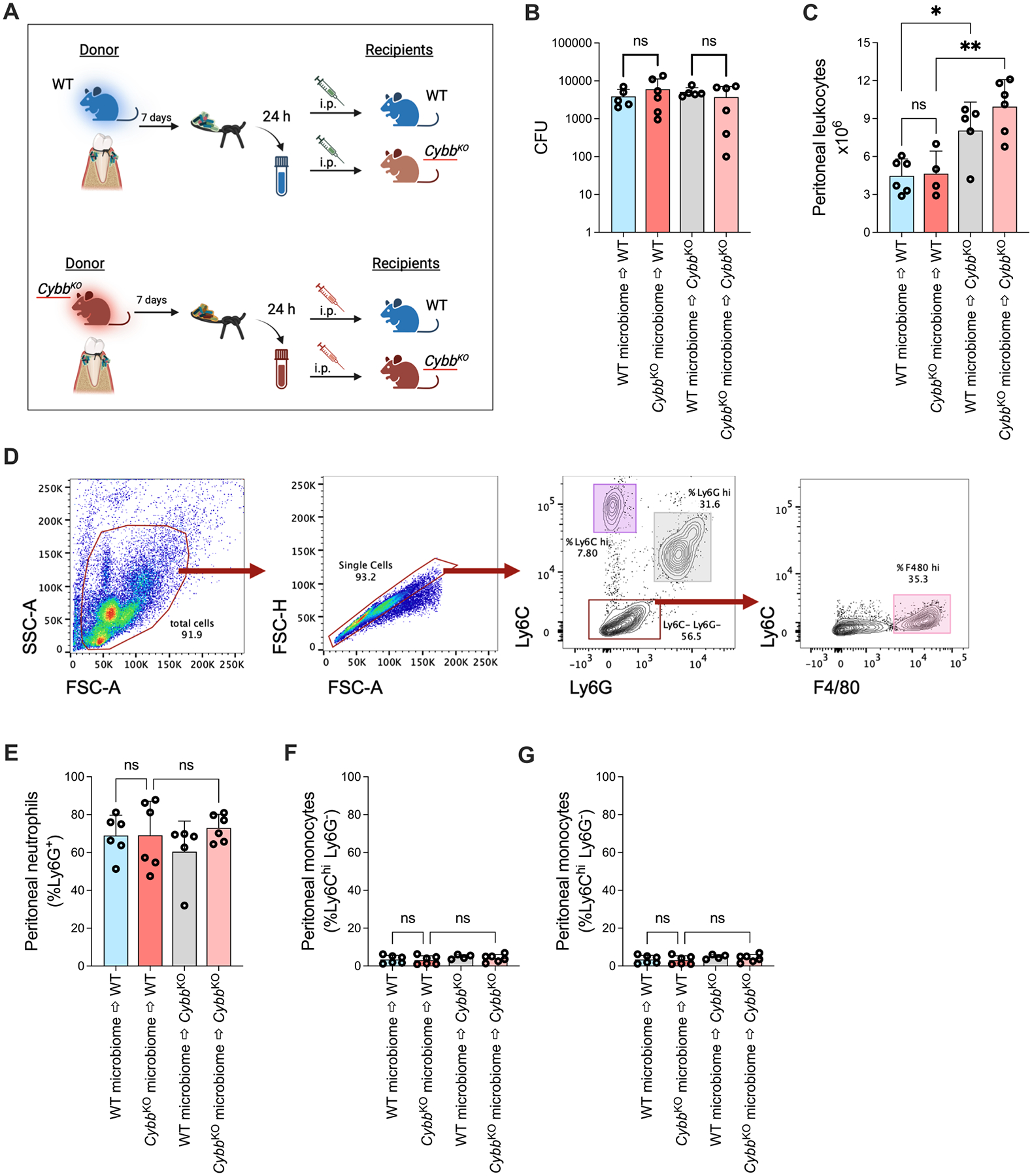
*Cybb*^*KO*^ clear oral bacteria with the same efficiency at WT mice: The ligature-associated microbiome was isolated on Day 8 from WT and *Cybb*^*KO*^ mice ‘donor’ mice and cultured overnight under aerobic and anaerobic conditions. The next day, 10^7^ pooled CFUs from aerobic and anaerobic overnight cultures were injected intraperitoneally in naïve or ‘recipient’ WT and *Cybb*^*KO*^ mice. After 4 h, peritoneal cavities were lavaged with sterile saline, and (B) peritoneal bacterial numbers (CFUs) were determined by plating on agar plates. (C)Total peritoneal cell numbers recovered. (D) Gating strategy for identification of peritoneal cells based on lineage markers. (E-G) Relative % of neutrophils (Ly6G^hi^ Ly6C^int^); monocytes (Ly6C^hi^ Ly6G^−^); and macrophages (F4/80^+^) in total peritoneal cells. Data from 3 mice per group are shown as mean ± SD, and statistical differences were determined using one-way ANOVA with Tukey’s multiple comparison test (*P < 0.05; **P < 0.01; ***P < 0.001; ****P < 0.0001).

Aerobic and anaerobic cultures of blood, draining lymph nodes, lungs, spleen, and liver homogenates from ligated WT and *Cybb*^*KO*^ mice were negative, indicating no evidence of sepsis or bacterial dissemination from the oral cavity. However, given the notable differences in the oral microbiomes of WT and *Cybb*^*KO*^ mice, we used a model of reciprocal microbiome transplant to determine relative virulence. The bacterial peritonitis model allows for quantitatively assessing antimicrobial killing capacity. Of note, the peritoneal cavity is sterile under naive conditions; thus, the introduction of bacteria elicits neutrophil and other inflammatory cell recruitment, allowing the profiling of relative bacterial killing across different strains, which is not possible at mucosal surfaces that are naturally populated with bacteria^36,37^. Pooled aerobic and anaerobic overnight cultures of ligature-associated bacteria from both strains were transplanted directly into the sterile peritoneal cavities of naïve WT and *Cybb*^*KO*^ mice as described in Fig. 4A. While we found no significant differences in relative bacterial clearance, as indicated by similar CFUs across all mice (Fig. 4B), *Cybb*^*KO*^ mice exhibited a more robust inflammatory response characterized by significantly higher neutrophil recruitment irrespective of the type of microbial challenge (Fig. 4C–G). These data indicate that the dysregulated inflammatory responses in *Cybb*^*KO*^ mice are not due to compromised antimicrobial capacity but are perhaps related to over-recruitment and/or hyperactivation of ROS-deficient neutrophils.

### Cybb^KO^ neutrophils exhibited dysregulated inflammatory responses to oral bacteria

**Fig. 5.**
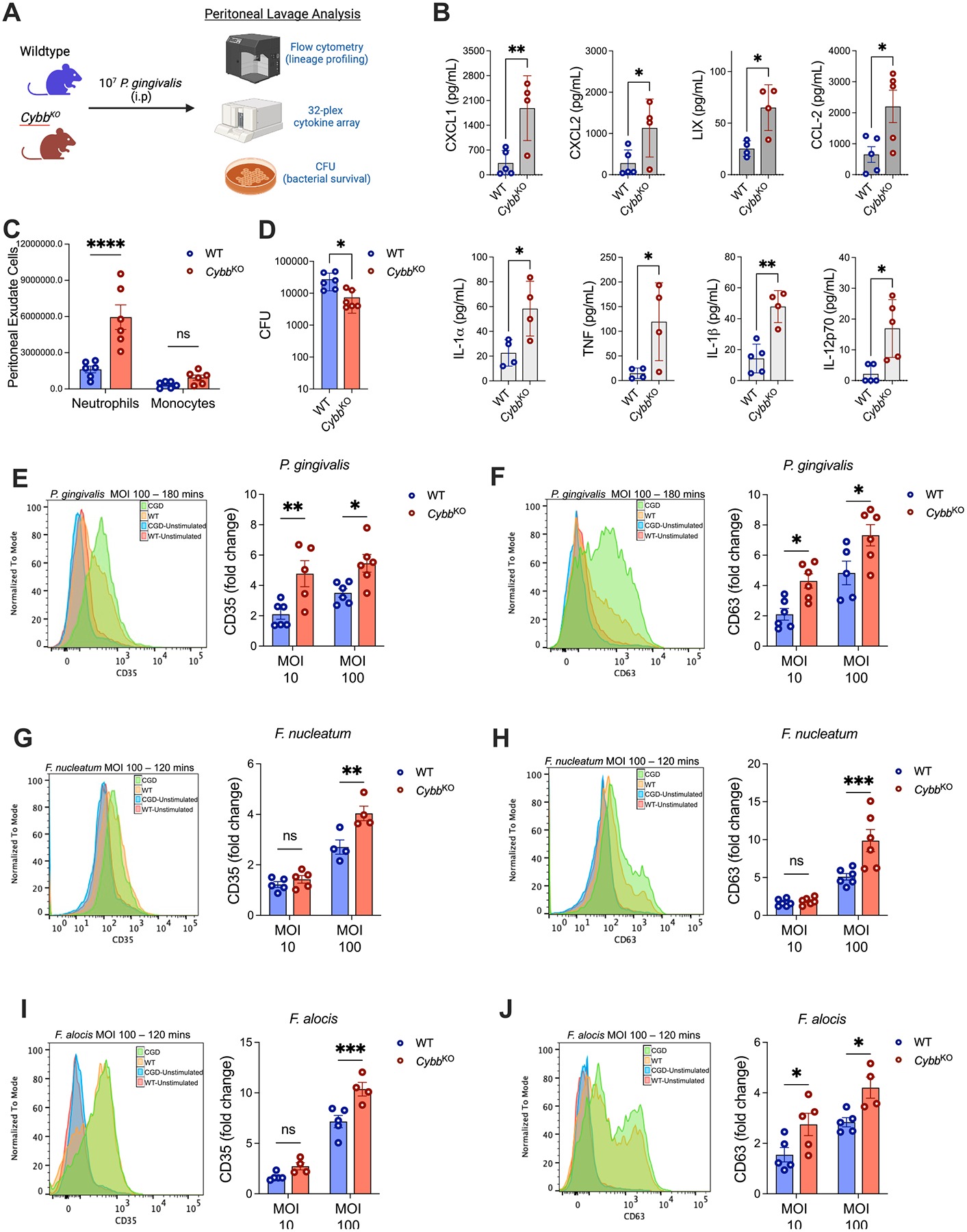
Nox2 deficiency drives excessive inflammatory cytokine production and degranulation in neutrophils. (**A**) Wild-type (WT) and *Cybb*^*KO*^ mice were intraperitoneally challenged with 10^7^ CFU of *P. gingivalis*. After 4 h, peritoneal cavities were lavaged, and peritoneal lavage fluid was analyzed. (**B**) Cytokine and chemokine levels in peritoneal lavage fluid. (**C**) Peritoneal cell numbers in lavage fluids were identified by flow cytometry, and neutrophils (Ly6G^hi^ Ly6C^int^) and monocytes (Ly6C^hi^ Ly6G^−^). (**D**) *P. gingivalis* numbers after 4 h were determined by plating on blood agar plates, and CFUs were estimated. (**E-J**) Bone marrow neutrophils (BMNs) from WT and *Cybb*^*KO*^ mice were challenged with oral bacteria at the indicated multiplicity of infection (MOI) for 2–3 h. Exocytosis of tertiary and azurophilic granules was quantified by measuring the surface abundance of CD35 and CD63, respectively by flow cytometry. % shifts are illustrated in histograms, and fold change from 3 to 7 mice (BMNs) is illustrated in adjacent bar graphs. Data from 4 to 6 mice per group are displayed as mean ± SD, and statistical differences were determined using a *t*-test or two-way ANOVA with Sidak’s multiple comparison test for grouped variables (*P < 0.05; **P < 0.01; ***P < 0.001; ****P < 0.0001).

The intensity of inflammatory responses in CGD is not always linked to active infection. In vivo challenges with bacterial ligands such as lipopolysaccharide^38^, fungal ligands (zymosan or hyphae)^17,18,39^, or ligands released during pyroptotic/necrotic cell death (ATP, monosodium urate crystals^16^) have all been linked to dysregulated neutrophil recruitment and several-fold higher activation of inflammatory genes compared to WT mice. Thus, we measured neutrophil effector responses to oral bacterial stimulation using the bacterial peritonitis model, which allows quantitative evaluation of multiple inflammatory responses to injected bacteria (Fig. 5A). We focused on *Porphyromonas gingivalis*, a relevant pathogen in human periodontal infections and highly adept at manipulating neutrophil effector responses. Four hours post i.p. challenge with 10^7^ CFU of *P. gingivalis, Cybb*^*KO*^ mice had significantly higher neutrophilic recruitment (Fig. 5C), which correlated with significantly elevated levels of neutrophil-attracting chemokines (CXCL1, CXCL2, and LIX), as well as pro-inflammatory cytokines (IL-1α, IL-1β, TNF, and IL-12p70) in the peritoneal lavage fluid (Fig. 5B). While we observed a significant increase in the monocyte chemoattractant CCL2, peritoneal monocyte numbers at 4 h post-challenge showed no difference (Fig. 5C). Transcriptional profiling of isolated peritoneal exudate neutrophils also suggested an increased presence of pro-inflammatory transcripts, indicating that Nox2-deficient neutrophils actively contribute towards establishing feed-forward inflammatory loops in a cell-intrinsic manner (Fig. S2A–C). Excessive inflammatory activation was also confirmed in purified naive bone marrow neutrophils (BMNs), in which ex vivo challenge with *P. gingivalis*, as well as other periodontal pathogens, led to significantly higher TNF production (Fig. S2D–F). Interestingly, our data demonstrate more efficient clearance of *P. gingivalis* in *Cybb*^*KO*^ peritoneal cavities, indicating that the dysregulated inflammatory response was not correlated with defective bacterial clearance (Fig. 5D). These observations corroborate our findings with the reciprocal oral microbiome challenge (Fig. 4) and are not limited to a single bacterium. In the context of *P. gingivalis*, we have previously established that Nox2 activation is not required for the intracellular killing of *P. gingivalis* by neutrophils^40^.

In addition to generating inflammatory cytokines, neutrophils undergo degranulation, releasing antimicrobial mediators and proteases that can damage tissues. Excessive neutrophil degranulation has been implicated in collateral tissue damage and chronic inflammation^41,42^. Neutrophils from patients with CGD hyper-degranulate in response to inflammatory cytokines and other agonists^10^. Thus, we compared relative degranulation responses of WT and *Cybb*^*KO*^ BMNs to oral periodontal pathogens. As expected, *Cybb*^*KO*^ BMNs exhibited significantly higher mobilization of primary (azurophilic) and tertiary (gelatinase) granules to the surface in response to *P. gingivalis* as well as other gram-negative (*Fusobacterium nucleatum*) and gram-positive (*Filifactor alocis*) periodontal pathogens (Fig. 5E–J). Thus, our data demonstrate that Nox2 deficiency led to broad dysregulation of neutrophil effector functions, resulting in excessive degranulation and pro-inflammatory cytokine production in response to the unbalanced microbiome that developed after ligature placement in mice. Next, we focused on identifying transcription factors driving excessive inflammatory cytokine responses in *Cybb*^*KO*^ mice.

### Neutrophilic oxygen consumption and Nrf2 activation are essential for limiting inflammatory damage at the oral mucosal barrier

At barrier surfaces, Nox2 activation is directly linked to the activation of redox-sensitive transcription factors that counter pro-inflammatory pathways and promote wound healing. For example, in inflamed colonic mucosa, transmigrating neutrophils undergo oxidative burst, inducing “inflammatory hypoxia” and the compensatory activation of hypoxia-induced transcription factor 1 alpha (HIF-1α) in epithelial cells, which then facilitates epithelial restitution^43^. Nox2 activation is also critical for activating another transcription factor, the nuclear factor-erythroid 2-related factor 2 (Nrf2), a master regulator of antioxidant responses. Nuclear translocation of Nrf2 and binding to the antioxidant response element in the promoter regions drive the transcription of multiple antioxidant genes. Independently, Nrf2, upon nuclear translocation, also *trans*-represses inflammatory transcription factors such as NF-κB^13,38^, thereby inhibiting the transcription of IL-6 and IL-1 independent of the ARE binding motif^44^.

In our RNA-seq data, we did not observe any differences in HIF-1α-regulated pathways, but we observed reduced Nrf2 (*Nfe2l2*) transcript levels in inflamed tissues of *Cybb*^*KO*^ mice (Fig. S3A). We further confirmed the expression *Nfe2l2* as well as Nrf2-regulated antioxidant genes (hemeoxygenase1 (*Hmox1*), NAD(P)H quinone dehydrogenase 1 (*Nqo1*), and Glutamate-cysteine ligase (*Gclc*)) by qPCR, indicating a broad suppression of Nrf2 activity in the oral tissues of *Cybb*^*KO*^ mice (Fig. S3B). Interestingly, our data are consistent with previous reports linking deficient Nrf2 activation in *Cybb*^*KO*^ mouse lungs to excessive NF-κB-driven hyperinflammation and pro-inflammatory cytokine expression. Therefore, we determined whether restoring Nrf2 activity using a synthetic agonist would ameliorate oral inflammation in *Cybb*^*KO*^ mice.

**Fig. 6.**
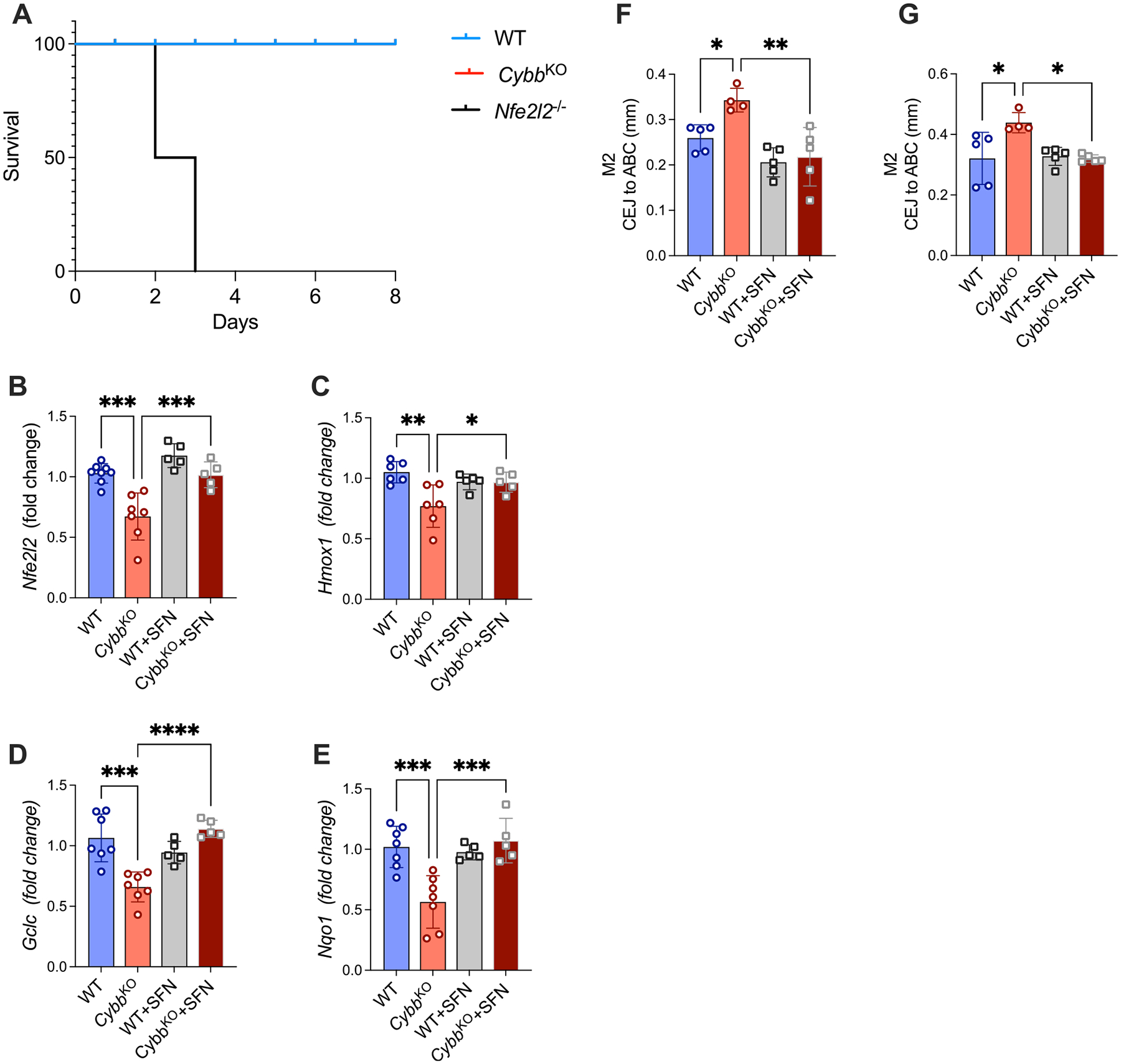
Insufficient activation of Nrf2 and downstream antioxidant responses exacerbate oral inflammation in Nox2-deficient mice. (**A**) Kaplan-Meier survival curve of wild-type (**WT**), *Cybb*^*KO*,^ and Nrf2 knockout (*Nfe2l2*^−/−^) mice during LIP. (**B-G**) WT and *Cybb*^*KO*^ mice were treated with 25 mg/kg sulforaphane daily for the entire duration of LIP. (**B-E**) Gingival tissues were extracted on Day 8, and **qPCR** analysis determined the relative expression of Nrf2-activated genes. (**F-G**) Average bone loss (in millimeters) in the interdental regions (M2, M2′) of the ligated molar and unligated contralateral control molars from the same mice. Data from 4 to 6 mice per group are displayed as mean ± SD, and statistical differences were determined using one-way ANOVA with Tukey’s multiple comparison test (*P < 0.05; **P < 0.01; ***P < 0.001; ****P < 0.0001).

In oral tissues, Nrf2 activation is crucial for protecting against oxidative damage, attenuating osteoclastogenesis, and promoting wound healing through the coordinated upregulation of antioxidant genes, thereby restoring cellular homeostasis^45–47^. Unlike WT and *Cybb*^*KO*^ mice, placing ligatures in Nrf2 knockout mice (*Nfe2l2*^−/−^) resulted in an early and severe inflammatory response, and mice had to be euthanized and removed from the study due to meeting the endpoint or humane criteria (Fig. 6A). These observations confirmed that failing to optimally activate Nrf2 in the oral mucosa could have devastating consequences. We used sulforaphane (SFN)^13,26^, a synthetic agonist for Nrf2, to restore Nrf2 activity and alleviate inflammatory bone loss in *Cybb*^*KO*^ mice. SFN treatment restored the significantly diminished expression of Nrf2 and Nrf2-regulated genes *Hmox1*, *Gclc*, and *Nqo1* (Fig. 6B–E) in the gingival tissues of inflamed *Cybb*^*KO*^ mice and considerably reduced alveolar bone recession to levels seen in WT mice (Fig. 6F–G).

## Discussion

In the oral cavity, neutrophils are essential for immune surveillance, and their efferocytic clearance is critical for activating resolution and anti-inflammatory pathways necessary for maintaining homeostasis and wound healing in oral tissues^48^. Congenital defects compromising neutrophil recruitment, such as those observed in Leukocyte Adhesion Deficiency 1 (LAD-1), severe congenital or cyclical neutropenias caused by neutrophil elastase (ELA2) deficiency, and Chédiak-Higashi syndrome, are all associated with severe periodontitis, often observed early in childhood^30,49,50^. Oral lesions and ulceration are also prevalent in Papillon-Lefèvre syndrome, caused by a deficiency of the lysosomal *exo*-cysteine protease cathepsin C, which is involved in the activation of neutrophil antimicrobial proteins cathepsin G, elastase, and proteinase 3. Neutrophils from patients with Papillon-Lefèvre syndrome have compromised antimicrobial capacity^49,50^. However, in contrast to other neutrophil-related immunodeficiencies, patients with CGD do not have abnormally low neutrophil counts, a compromised granule repertoire, or defects in neutrophil recruitment to peripheral tissues such as the oral mucosa. Thus, the molecular mechanisms underlying oral complications in CGD are distinct from those in other genetic defects that affect neutrophil function.

Oral inflammation, particularly periodontitis, is an infection-driven disease in which microbial virulence factors actively thwart immune clearance and perpetuate inflammation^51,52^. Coupled with the higher incidence of infection in CGD, one would expect that the hyperinflammatory responses in *Cybb*^*KO*^ mice were linked to poor antimicrobial clearance. However, our findings challenge this notion and show that the increased susceptibility of *Cybb*^*KO*^ mice to periodontitis was attributed to dysregulation of the host inflammatory response, specifically to excessive recruitment and overactivation of ROS-deficient neutrophils. Furthermore, Nox2-deficient neutrophils cannot form neutrophil extracellular traps or mount an oxidative burst, thus making them inefficient at trapping and eliminating microbes^9^. However, clinically, patients with CGD exhibit susceptibility to a very limited spectrum of bacteria, indicating that other neutrophil antimicrobial mechanisms, such as the release of antimicrobial peptides and serine proteases, are effective in inactivating a wide variety of pathogens and also protect against endogenous colonizers or commensals^35^. We do not observe any naturally occurring bone loss in naïve *Cybb*^*KO*^ mice, further confirming that Nox2 deficiency does not naturally predispose to infections from oral pathobionts. Interestingly, we observed differences in the microbial composition of ligature-associated biofilm bacteria within the two strains under inflammatory conditions. It is likely that these differences arise from the enrichment of pathogenic species that produce virulence factors such as leukocidal toxins, proteases, or other virulence factors that enable them to survive the neutrophil onslaught. Many periodontal pathogens thrive in a chronic inflammatory, neutrophil-dominated subgingival environment due to their ability to actively impair neutrophil killing. For example, *P. gingivalis* protease RgpB can degrade neutrophil azurophilic granule proteases, thereby prolonging intracellular survival^40,53^. In our microbiome transplant experiments, we did not observe any defect in the killing of oral biofilm pathogens by Nox2-deficient neutrophils compared with WT neutrophils. Instead, we found excessive production of pro-inflammatory cytokines from neutrophils with absent ROS responses. These observations, coupled with the absence of systemic spread of oral microbes, argue against sub-optimal microbial clearance as an overwhelming driver of oral inflammation in *Cybb*^*KO*^ mice.

A few ex vivo studies have shown excessive ROS generation by neutrophils isolated from the peripheral blood of patients with periodontitis and have concluded that oxidative stress resulting from hyperactivation of Nox2 is a significant contributing factor to the pathophysiology of periodontitis^54,55^. However, given the elevated systemic inflammatory status of patients with periodontitis, it is difficult to determine whether Nox2 activity was a cause or consequence of neutrophil activation and inferred periodontal tissue destruction. Another significant confounding factor is the use of chemical inhibitors of ROS, such as N-acetylcysteine, that non-specifically inhibit multiple inflammatory pathways, including c-Jun N-terminal kinase, p38 MAP kinase, and NF-κB, thus complicating the interpretation of these studies^56^. In contrast to these papers, our studies relied on a precise genetic deletion of Nox2 activity to demonstrate that the Nox2-derived ROS are, in fact, necessary to provide negative feedback that restrains neutrophil hyperactivation in the context of oral inflammation, damage, and dysbiosis. Our findings align with several other reports that confirm the newly emerging role of Nox2 in restraining the magnitude of host inflammatory responses^9,19,57^. Nox2 activity was essential for activating Nrf2 and Nrf2-mediated downregulation of the oral inflammatory response. Future studies will investigate whether Nrf2 restrains neutrophil-specific responses or impacts multiple cell types within oral tissues to subdue inflammation.

## Supplementary Material

1

## Appendix A. Supplementary data

Supplementary data to this article can be found online at https://doi.org/10.1016/j.mucimm.2026.04.002.
